# Mapping the transcriptomics landscape of post-traumatic stress disorder symptom dimensions in World Trade Center responders

**DOI:** 10.1038/s41398-021-01431-6

**Published:** 2021-05-24

**Authors:** Pei-Fen Kuan, Xiaohua Yang, Xu Ren, Chang Che, Monika Waszczuk, Roman Kotov, Sean Clouston, Prashant K. Singh, Sean T. Glenn, Eduardo Cortes Gomez, Jianmin Wang, Evelyn Bromet, Benjamin J. Luft

**Affiliations:** 1 Department of Applied Mathematics and Statistics, Stony Brook University, Stony Brook, NY USA; 2 Department of Medicine, Stony Brook University, Stony Brook, NY USA; 3 Department of Psychology, Rosalind Franklin University of Medicine and Science, North Chicago, IL USA; 4 Department of Psychiatry, Stony Book University, Stony Brook, NY USA; 5 Department of Family and Preventive Medicine, Stony Book University, Stony Brook, NY USA; 6 Department of Cancer Genetics, Roswell Park Cancer Institute, Buffalo, NY USA; 7 Department of Biostatistics and Bioinformatics, Roswell Park Cancer Institute, Buffalo, NY USA

**Keywords:** Genomics, Biomarkers

## Abstract

Gene expression has provided promising insights into the pathophysiology of post-traumatic stress disorder (PTSD); however, specific regulatory transcriptomic mechanisms remain unknown. The present study addressed this limitation by performing transcriptome-wide RNA-Seq of whole-blood samples from 226 World Trade Center responders. The investigation focused on differential expression (DE) at the gene, isoform, and for the first time, alternative splicing (AS) levels associated with the symptoms of PTSD: total burden, re-experiencing, avoidance, numbing, and hyperarousal subdimensions. These symptoms were associated with 76, 1, 48, 15, and 49 DE genes, respectively (FDR < 0.05). Moreover, they were associated with 103, 11, 0, 43, and 32 AS events. Avoidance differed the most from other dimensions with respect to DE genes and AS events. Gene set enrichment analysis (GSEA) identified pathways involved in inflammatory and metabolic processes, which may have implications in the treatment of PTSD. Overall, the findings shed a novel light on the wide range of transcriptomic alterations associated with PTSD at the gene and AS levels. The results of DE analysis associated with PTSD subdimensions highlights the importance of studying PTSD symptom heterogeneity.

## Introduction

Post-traumatic stress disorder (PTSD) is a complex condition arising in the wake of exposure to a traumatic event, and the lifetime prevalence of PTSD in the U.S. is 6.1%^1^. PTSD is heterogeneous and characterized by symptoms of intrusive re-experiencing of the trauma (e.g., flashbacks), avoidance of reminders of the trauma, emotional numbness and negative effects, and increased arousal^2^. Many patients with PTSD are treatment refractory^3^, placing them at risk of developing chronic physical conditions and long-term cognitive, social, and occupational impairments^4^, thus imposing considerable socioeconomic burden^5^. As a result, there is a critical need to understand the biological processes that underpin and maintain PTSD with a view to identifying novel biomarkers to aid in diagnosis and monitoring, and ultimately in the discovery of therapeutic targets.

Increasing evidence from epidemiologic and genetic studies shows that genetic factors and environmental exposure play important roles in the etiology of PTSD^6–9^. In particular, differential gene expression, which captures the effects of both genetic and environmental influences, has emerged as a crucial biological process implicated in vulnerability to PTSD. Thus, gene expression can serve as a promising biomarker to understand the pathophysiological mechanisms of PTSD and may prove to be involved in both the etiology and progression of the disorder. Gene expression is a complex process encompassing transcription, RNA splicing, translation, and post-translational modification^10^. However, to date, most PTSD biomarker studies, including those conducted by our group, have focused on identifying differential gene expression associated with PTSD only at the gene level using transcriptomics technologies, such as microarrays and RNA-sequencing (RNA-Seq)^11–14^. These studies have identified differentially expressed (DE) genes that play a role in the regulation of the glucocorticoid (GC) receptor in the GC signaling pathway, neuronal signaling, and immune responses to stress^12,14–18^.

**Fig. 1:**
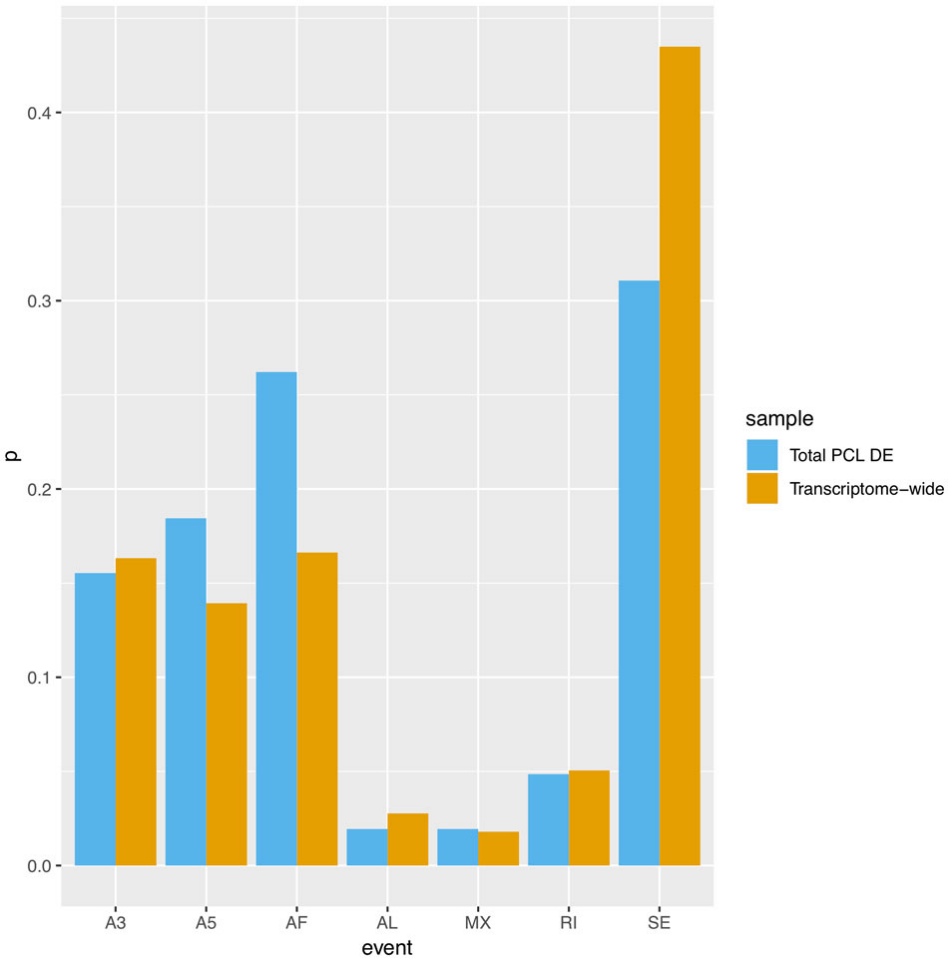
Bar graph comparing the proportions of significant AS events associated with total PCL to the transcriptome-wide proportions of AS events detected by SUPPA. *A3* alternative 3’ splice sites, *A5* alternative 5’ splice sites, *AF* alternative first exons, *AL* alternative last exons, *MX* autually exclusive exons, *RI* retained introns, *SE* skipping exons.

RNA-Seq has emerged as the state-of-the-art platform for transcriptomics profiling since it offers a broader dynamic range than microarrays and allows for the detection of low-abundance transcripts^19^. Existing PTSD studies utilizing RNA-Seq to quantify expression at the gene level do not extend to the detection of splice variant/isoform differences and novel transcripts. Alternative splicing (AS) is an important regulatory mechanism that increases the functional capacity of a gene. Examples of AS events include exons or introns of a gene within a pre-mRNA transcript that are differentially joined or skipped (i.e., skipping exons, mutually exclusive exons, and retained introns), resulting in multiple protein isoforms being encoded by a single gene. Other types of splicing events include alternative 5’ and 3’ splice sites (see Fig. 1B of Alamancos, Pages^20^ for a description of these AS events). Changes in AS have been shown to contribute to lymphocyte functions during the immune response and to regulate T-cell responses to antigens^21–23^. These studies provide evidence of AS in T-cell activation and B-cell stimulation. The gene set regulated by AS does not display significant changes at the gene level^22^. Since immune response genes have been implicated in PTSD, expanding transcriptomics research beyond the gene level to splice variant-level analysis could unravel new biological mechanisms contributing to the maintenance of PTSD over time.

Another crucial and previously unexplored issue in gene expression studies is the fact that PTSD is a heterogeneous disorder with marked variability in symptom presentation, etiology, severity, and persistence^5^. Multiple models have been proposed; nevertheless, the model that has received the most support to date in trauma-exposed samples specifies four dimensions within DSM-IV PTSD: (1) re-experiencing (e.g., flashbacks of the traumatic event, intrusive dreams); (2) avoidance; (3) numbing; and (4) hyperarousal^24,25^. Twin studies have found that PTSD dimensions are characterized by distinct genetic patterns^8^, suggesting the presence of unique downstream genetic pathways characterizing each dimension. This is consistent with emerging molecular genetic evidence; for example a recent genome-wide association study identified multiple SNPs associated with the risk of certain PTSD subscales but not others^26^. Furthermore, our team previously found that polygenic risk scores for PTSD and comorbid psychiatric conditions show a degree of differential association with PCL subscales^27^. Finally, studies of immune system biomarkers have identified a specific link between PTSD-related avoidance and inflammation^28,29^. Taken together, these results are consistent with a growing body of evidence that PTSD dimensions are associated with separate biological processes^30,31^, and it is plausible that PTSD dimensions are characterized by different gene expression patterns. Nonetheless, all prior gene expression association studies, including those conducted by our group, conceptualize PTSD as a unitary disorder and none examine transcriptomic patterns specific to PTSD dimensions^12,14,32^.

The purpose of the present study was to investigate the associations of gene expression with the overall severity of PTSD symptoms and its four dimensions using RNA-Seq to evaluate patterns at the gene, isoform, and AS levels. To this end, we analyzed whole-blood samples from 226 World Trade Center (WTC) responders who were exposed to the same disaster, the 9/11 attacks in New York City, thus minimizing heterogeneity of the trauma.

## Methods

### Participants and PTSD assessment

Participants were recruited through the Stony Brook WTC-Health Program^33^. The present study was approved by Stony Brook University IRB and written informed consent was obtained from all participants. Inclusion criteria were adequate English language skills to complete the protocol and being male. We included only males because females show notably different gene expression patterns from males^34^, and only <10% of responders in the Stony Brook cohort were female. Data were collected during 2014–2016, which was 13–15 years after the WTC collapse. PTSD symptom severity (total and four dimensions, namely re-experiencing, avoidance, numbing, and hyperarousal) was measured using the Post-traumatic Stress Disorder Checklist-Specific Version (PCL-17)^35^, a 17-item self-report questionnaire modified to assess the severity of DSM-IV WTC-related PTSD symptoms over the past month on a scale of 1 (never bothered by) to 5 (extremely bothered by) (Cronbach’s *α* = 0.96). Our research team has previously validated the four-dimensional model in WTC responders and found it to be more informative than the alternatives^36,37^. The analytical sample included 226 participants (an independent cohort from our previous gene expression study^12^). All participants were male; 84.5% were Caucasian; and the mean age was 52.67 years old (*SD* = 8.02).

### RNA-Seq data preprocessing

Gene expression in whole blood was profiled at the Genomics Shared Resource of Roswell Park Comprehensive Cancer Center. The whole transcriptome libraries were prepared using KAPA HyperPrep Kit with RiboErase (HMR) kit (Roche Sequencing Solutions) and sequenced on Illumina NovaSeq 6000 sequencer at a sequencing depth of over 100 million paired end reads (100 bp) per sample. Raw reads that passed the quality filter from Illumina RTA were pre-processed using fastqc^38^ for sequencing base quality control and cutadapt^39^ to remove adapter sequences if applicable. Alignment was performed with the TopHat2 software^40,41^, utilizing Bowtie2 (http://bowtie-bio.sourceforge.net/bowtie2/index.shtml) in the RefSeq (NCBI Reference Sequence Database) annotation database^42^ and the human reference genome (GrCh37-hg19 version). Other genomic-related data were obtained using the UCSC genome repository^43,44^. A second round of QC using RSeQC was applied to the mapped bam files to identify potential RNA-Seq library preparation problems. From the mapping results, the number of reads aligned to each gene was calculated using HTSeq^45^. The raw count data were transformed into fragments per kilobase of transcript per million mapped reads (FPKM) and transcripts per million mapped reads (TPM) to account for library size differences across the samples.

### Isoform-level and alternative splicing quantification

Isoform-level quantification was performed with the Salmon software^46^, whereas the SUPPA software^20^ was used to quantify the different AS events, namely skipping exons, alternative 5’/3’ splice sites, mutually exclusive exons, retained introns, and alternative first/last exons. Each AS event was represented as a PSI_ori_ value, defined as the ratio of abundance of transcripts that include the exon over the abundance of transcripts that include or skip the exon. PSI = log(PSI_ori_/(1-PSI_ori_)) was used for AS quantification in our analysis. Genes and isoforms with low read counts were filtered out prior to statistical analysis.

### Estimation of batch effects

The potential for batch effects was estimated using the surrogate variable analysis approach for sequencing data (svaseq)^47^. Proportions of CD4T, CD8T, monocytes, natural killer (NK), B-cells, macrophage, dendritic, mast cells, eosinophils, and neutrophils were estimated using the CIBERSORT software^48^. The correlations between the estimated proportions of cell types and PCL were compared using Pearson correlation coefficients. The estimated surrogate variables and proportion of cell types were included as covariates in the DE analysis.

### Differential expression analysis

DE analysis of gene-level count data was carried out using NBAMSeq^49^ to identify genes associated with total PCL and each dimension, following adjustment for age, race, cell proportions (CD4T, CD8T, monocytes, NK, and B-cells), and potential surrogate variables. NBAMSeq is a recently developed method by our group for RNA-Seq analysis based on a flexible generalized additive model that enables the detection of both linear and nonlinear associations between gene expression and the phenotype of interest^49^.

DE analysis at the isoform and AS levels was performed with isoform log(TPM + 1) and PSI, respectively, to identify events associated with total PCL and its dimensions using spline regression^50^, following adjustment for age, race, cell proportions, and potential surrogate variables.

A false discovery rate (FDR)^51^ < 0.05 was used to identify statistically significant DE genes. Statistically significant genes with an estimated effective degree of freedom (edf) > 1.5 were considered nonlinear DE genes^49^. The edf can be regarded as a proxy for the degree of nonlinearity, where edf = 1 implies that the function reduces to a linear effect model, whereas a large edf implies greater deviation from the linear effect model. Among the nonlinear DE genes, *post-hoc* analysis was conducted on the estimated smooth functions of total PCL to characterize the nonlinear patterns. Specifically, the estimated functions of these genes were clustered via k-medoid clustering^52^, and the optimal number of clusters was determined using gap statistics^53^. The top genes and AS events unique to each dimension were defined at FDR < 0.05 for a target dimension and *p* > 0.1 for the other three dimensions. For example, the top genes unique to re-experiencing were defined as those with FDR < 0.05 in re-experiencing DE analysis and *p* > 0.1 in avoidance, numbing, and hyperarousal DE analyses.

The proportions of different AS events identified by DE analysis were compared with the transcriptome-wide proportions detected by SUPPA using the chi-square goodness-of-fit test, in which categories with expected counts <5 were combined.

### Gene set enrichment analysis (GSEA)

For each PCL dimension DE analysis result, GSEA^54^ was conducted on the entire list of genes ranked by negative log *p* values. Both the gene ontology (GO)^55^ and KEGG canonical pathway^56^ gene sets were tested. The minimum and maximum gene set sizes were 15 and 500. FDR < 0.1 was used to identify statistically significant gene sets for each comparison. If no pathway was significant at FDR < 0.1 (quantified by a *q* < 0.1^57^), the gene sets at *p* < 0.001 were reported. The top gene sets unique to each dimension were defined at FDR < 0.1 or *p* < 0.001 for a target dimension, and *p* > 0.1 for the other three dimensions. For example, the top gene sets unique to re-experiencing were defined as those with either FDR < 0.1 or *p* < 0.001 in re-experiencing DE analysis and *p* > 0.1 in avoidance, numbing, and hyperarousal DE analyses.

For each list of genes unique to each dimension from the DE analysis, the DAVID functional annotation tool (https://david.ncifcrf.gov/) was used to identify enriched pathways. Pathways significant at FDR < 0.1 were reported.

Additional statistical analyses based on the weighted gene co-expression network analysis (WGCNA)^58^ to identify modules of correlated genes, isoforms and AS events were provided in Supplementary Materials.

## Results

### DE genes, isoforms, and AS events associated with total PTSD

The mean total PCL score was 36.59 (*SD* = 15.45). Estimated cell proportions were not associated with total PCL (| r | < 0.105, *p* > 0.11 for each cell proportion).

DE analysis identified 76 genes associated with total PCL at FDR < 0.05. A total of 70 out of the 76 genes showed nonlinear associations with total PCL, with an estimated edf ranging from 1.75 to 4.22 (mean edf: 2.54). DE genes and the estimated edf are provided in Supplementary Table 1. *Post-hoc* clustering analysis of the estimated smooth functions of total PCL for the 70 nonlinear DE genes identified 5 as the optimal number of clusters (Supplementary Fig. 1A). Supplementary Fig. 1B–F shows the five identified clusters. Within each cluster, the gray lines correspond to the estimated smooth functions of individual genes, whereas the red line corresponds to the mean estimated smooth function of the genes in the cluster. DE genes and the cluster membership are provided in Supplementary Table 1.

Next, 12,071 AS events were detected in 6528 genes using SUPPA and were divided into seven types: A3 alternative 3’ splice sites, A5 alternative 5’ splice sites, AF alternative first exons, AL alternative last exons, MX mutually exclusive exons, RI retained introns, and SE skipping exons. Skipping exons constituted the largest number of AS events, at 5250. The relative proportions of each event are given in Fig. 1. The *UTY* gene contained the largest number of AS events in our dataset, consistent with previous findings that this gene has a huge splicing frequency^59^. At FDR < 0.05, 103 AS events were associated with total PCL. A total of 101 out of the 103 identified events had nonlinear associations with total PCL. The relative proportions of the seven event types are shown in Fig. 1, which suggest that the proportion of alternative first exons (AF) was higher, whereas the proportion of skipping exons (SE) was lower as compared with transcriptome-wide proportions (chi-square test *p* < 0.05 after combining AL, MX, and RI). The top AS events included A5 and SE in the *BTNL8* gene, in addition to AF and A3 in the *BANP* gene (Supplementary Table 2).

No isoform was significantly associated with total PCL at FDR < 0.05. The smallest FDR was 0.058, which corresponded to isoform NM_001143760, *EIF5A* transcript variant A.

### Gene set enrichment analysis (GSEA) associated with total PTSD

GSEA identified 45 GO terms and 26 canonical pathways associated with total PCL (Supplementary Table 3). The top GO terms included interleukin-17 production and response to type I interferon, whereas the top canonical pathways included nervous system development and interferon-α/β signaling.

### DE genes, isoforms, and AS events associated with PTSD dimensions

The pairwise correlations among the four dimensions ranged from 0.62 to 0.84 (Supplementary Fig. 2A). On the other hand, comparison between PCL and cell proportions did not yield significant correlations (Supplementary Fig. 2B).

DE analysis identified 1, 48, 15, and 49 significant genes for re-experiencing, avoidance, numbing, and hyperarousal, respectively, at an FDR < 0.05. More than 83% of the DE genes showed a nonlinear association with the phenotypes, with an estimated edf ranging from 1.6 to 7.1 (mean edf: 2.7). DE genes and the estimated edf are provided in Supplementary Table 1. All the genes identified by re-experiencing, numbing, and hyperarousal had *p* < 0.1 in the total PCL analysis. On the other hand, 21 out of the 48 genes associated with avoidance had *p* > 0.1 in the total PCL analysis (Supplementary Table 1).

**Fig. 2:**
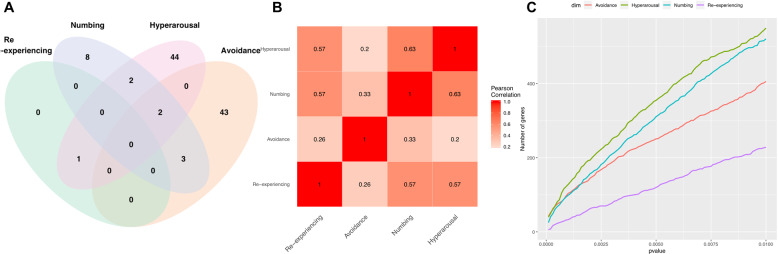
Comparisons of DE genes associated with each PTSD dimension. **A** Venn diagram comparing the overlap among genes associated with re-experiencing, avoidance, numbing, and hyperarousal. **B** Pearson correlation coefficients comparing the negative log *p* values among re-experiencing, avoidance, numbing, and hyperarousal. **C** Number of significant genes at different *p* value thresholds.

The number of overlapping genes among the four dimensions is reported in Fig. 2A. Pearson correlation coefficients computed for the estimated negative log *p* values from NBAMSeq to summarize the strength of gene expression association among re-experiencing, avoidance, numbing, and hyperarousal are given in Fig. 2B, which indicate that DE analysis of avoidance had the lowest correlation as compared with that of the other dimensions. A total of 23 and 2 genes were unique to avoidance and hyperarousal DE analysis, respectively, whereas no genes were unique to re-experiencing or numbing (Table 1). Across the *p* value thresholds, hyperarousal and re-experiencing had the most and least number of significant genes, respectively (Fig. 2C).

**Table 1:** List of DE genes and AS events unique to each PCL dimension analysis.

Dimension	Unique DE genes	Unique AS events
Re-experiencing	None	*SIGLEC10*:A3, *UPF2*:AF, *POLD3*:SE
Avoidance	*AHSP*, *ALAS2*, *CA1*, *CCNDBP1*, *CMAS*, *CREG1*, *CTNNAL1*, *DPCD*, *GABARAPL2*, *GLRX5*, *H2AFJ*, *HBD*, *HMBS*, *PAGE2B*, *PCTP*, *PITHD1*, *POLR1D*, *PRDX2*, *RGCC*, *RRAGA*, *SLC22A4*, *TERF2IP*, *TFRC*	None
Numbing	None	*ATP2A3*:A3, *HAUS4*:A5, *RNPS1*:AF, *RAD51B*:AL
Hyperarousal	*LOC728743*, *STMN3*	None

*A3* alternative 3’ splice sites, *A5* alternative 5’ splice sites, *AF* alternative first exons, *AL* alternative last exons, *MX* mutually exclusive exons, *RI* retained introns, *SE* skipping exons.

Only 1 isoform (NM_017668, *NDE1* transcript variant 2) was detected in hyperarousal DE analysis, while none were detected for re-experiencing, avoidance, or numbing at FDR < 0.05. Global results at the isoform level mimicked the overall trend observed at the gene level, where DE analysis of avoidance had the lowest correlation as compared with that of the other dimensions (Supplementary Fig. 3A). At different *p* value thresholds, numbing had the largest number of significant genes, followed closely by hyperarousal; re-experiencing was intermediate and avoidance had the lowest signals in isoform-level analysis (Supplementary Fig. 3B).

Finally, 11, 0, 43, and 32 AS events were associated with re-experiencing, avoidance, numbing, and hyperarousal, respectively, at FDR < 0.05. All the AS events associated with re-experiencing and hyperarousal showed nonlinear associations, whereas 42 out of the 43 AS events had nonlinear associations with numbing. The proportion of alternative first exons (AF) associated with hyperarousal was higher, whereas the proportion of skipping exons (SE) associated with both hyperarousal and numbing was lower as compared with transcriptome-wide proportions (chi-square test *p* < 0.05 after combining AL, MX, and RI) (Supplementary Fig. 3C). In total, 3 and 5 AS events were unique to re-experiencing and numbing DE analysis, respectively, whereas no events were unique to hyperarousal (Table 1). Global results at the AS level also mimicked the trend observed at the gene and isoform levels, where DE analysis of avoidance had the lowest correlation as compared with that of the other dimensions (Supplementary Fig. 3D). At different *p* value thresholds, numbing had the largest number of significant AS events, followed by hyperarousal; whereas, re-experiencing and avoidance had lower signals in AS-level analysis (Supplementary Fig. 3E). DE AS events are provided in Supplementary Table 2.

### Gene set enrichment analysis (GSEA) associated with PTSD dimensions

GSEA identified 27, 31, 32, and 49 GO gene sets associated with re-experiencing, avoidance, numbing, and hyperarousal, respectively (Supplementary Table 3). All the GO terms identified by numbing and hyperarousal had *p* < 0.1 in the total PCL analysis. On the other hand, 3 out of the 27 GO terms associated with re-experiencing and 18 out of the 31 GO terms associated with avoidance had *p* > 0.1 in the total PCL analysis (Supplementary Table 3). Comparison of the GSEA results of the four dimensions identified 4, 15, 5, and 4 GO terms unique to re-experiencing, avoidance, numbing, and hyperarousal, respectively (Table 2).

**Table 2:** List of GO and canonical pathway gene sets unique to each PCL dimension analysis.

Dimension	Unique GO gene sets	Unique canonical pathways
Re-experiencing	Positive regulation of chemotaxis Positive regulation of cyclin dependent protein kinase activity Positive regulation of dephosphorylation Regulation of chemotaxis	Matrisome
Avoidance	Antibiotic metabolic process Antioxidant activity Autophagosome Cellular detoxification Cofactor catabolic process Cofactor metabolic process Hydrogen peroxide metabolic process Organelle disassembly Oxidoreductase activity acting on peroxide acceptors Regulation of TOR signaling Response to starvation Tetrapyrrole biosynthetic process Tetrapyrrole metabolic process TOR signaling Transcription coactivator activity	Energy-dependent regulation of *mTOR* by *LKB1–AMPK*
Numbing	Catalytic step 2 spliceosome Protein targeting to mitochondrion Regulation of response to cytokine stimulus Spliceosomal complex U2 type spliceosomal complex	Spliceosome
Hyperarousal	Cytosolic transport Ribosomal RNA binding Polysomal ribosome miRNA metabolic process	Phosphoinositide pathway RAS pathway

GSEA identified 4, 5, 9, and 26 canonical pathways associated with re-experiencing, avoidance, numbing, and hyperarousal, respectively (Supplementary Table 3). All the canonical pathways identified by numbing and hyperarousal had *p* < 0.1 in the total PCL analysis. On the other hand, one out of the four pathways associated with re-experiencing and two out of the five pathways associated with avoidance had *p* > 0.1 in the total PCL analysis (Supplementary Table 3). Comparison of the GSEA results of the four dimensions identified 1, 1, 1, and 2 canonical pathways unique to re-experiencing, avoidance, numbing, and hyperarousal, respectively (Table 2). Global results at the GSEA level are consistent with those at the gene and isoform levels, where GSEA of avoidance had the lowest correlation as compared with the other dimensions for both GO terms and canonical pathways (Supplementary Fig. 4A–B). In addition, among the 23 genes unique to avoidance, the hemoglobin chaperone pathway (Biocarta), acetylation, and cytosol GO terms were significant at FDR < 0.1.

Additional analyses based on WGCNA^58^ showed that most of the statistically significant modules had nonlinear associations with total PCL and the dimensions (Supplementary Table 4, Supplementary Fig. 5). These modules were associated with several immune related ontologies, including neutrophil activation, interferon signaling pathway and viral response (Supplementary Table 5). The results also indicated that analysis of avoidance had the lowest correlation as compared with that of the other dimensions (Supplementary Fig. 6A, C, E). Comparisons of the number of significant module eigen-gene, eigen-isoform, and eigen-AS at different *p* value thresholds were shown in Supplementary Fig. 6B, D, F. Additional details were provided in Supplementary Materials.

## Discussion

The present study is the first to compare differential expression patterns at the gene, isoform, and AS levels associated with overall PTSD symptoms and its four dimensions. *IFI17* (interferon-α-inducible protein 27), a gene involved in innate immunity, was identified as the top gene associated with total PCL. *IFI27* has previously been found to be associated with chronic exposure to adverse social environments^60^. The analysis of downstream biological pathways enriched by DE genes revealed that the top GO terms and canonical pathways for total PCL included interleukin-17 production, interferon-α/β signaling, and response to type I interferon. These findings are consistent with the current literature showing that PTSD is associated with inflammation and the immune response^12–14^. The type I interferon pathway has also previously been found to be a shared inflammatory pathway across multiple modes of trauma in a transcriptome mega-analysis^14^.

Our results demonstrate a pattern of distinct yet intercorrelated DE associated with each of the four dimensions, adding to the growing body of evidence that unique biological pathways underpin the disorder subtypes. Specifically, the largest gene expression difference was between the avoidance dimension and the other PCL dimensions. The 23 DE genes unique to avoidance were enriched in the hemoglobin chaperone pathway, acetylation, and cytosol GO terms. Furthermore, three DE genes unique to avoidance, namely *AHSP*, *ALAS*, and *HMBS*, were involved in the hemoglobin chaperone pathway. Hemoglobin is responsible for delivering oxygen to tissues, and *AHSP* is a molecular chaperone that prevents its precipitation, thereby acting as a balancing component of hemoglobin^61^. The biological mechanism underlying hemoglobin regulation in PTSD is unclear; however, some studies have established an association between depression/anxiety disorders and hemoglobin levels and anemia^62,63^. GSEA identified energy-dependent regulation of *mTOR* by the *LKB1–AMPK* pathway unique to avoidance. Both the *AMPK* and *mTOR* serine/threonine kinases were involved in growth control, cell proliferation, and metabolism^64^. In addition, several of the GO terms unique to avoidance were involved in metabolic processes. This observation is interesting given that PTSD has been found to be a risk factor for metabolic syndromes^65,66^, suggesting that further investigation of the molecular and cellular mechanisms associated with the avoidance dimension may provide important insights into metabolic problems in PTSD.

In contrast to previous studies, the present study utilized transcriptome-wide AS quantification and identified 103, 11, 0, 42, and 32 AS events associated with total PCL, re-experiencing, avoidance, numbing, and hyperarousal, respectively. AS is a process by which gene diversity is increased. Among the DE AS events associated with total PCL, the proportions of AF and SE were different from transcriptome-wide proportions. Emerging evidence shows that variability in the human immune response is associated with differential splicing and isoform usage^67,68^, suggesting that the DE AS events identified in the present study could contribute to the link between PTSD and the inflammatory and immune responses. Specifically, differential SE and alternative 5’ splice sites in the *BTNL8* gene were among the top DE AS events associated with total PCL. *BTNL8* is involved in the primary immune response and co-stimulates T-cell proliferation and cytokine production^69^. This gene was not significantly associated with total PCL in gene-level analysis, indicating that analysis at the AS level could provide additional insights into gene regulation. In addition, across different *p* value thresholds, the numbing association analysis identified the largest number of AS events as compared with other PCL dimensions. This result is consistent with the GSEA analysis, in which the gene sets unique to the numbing dimension were involved in the spliceosome, a large molecular complex that catalyzes the splicing process^70^.

Last, among the DE genes in PTSD, many showed a nonlinear association with the total PCL and each dimension, suggesting that future research on gene expression and quantitative measurements of psychopathology (i.e., continuous) could potentially gain power by exploring nonlinear patterns.

### Strengths and limitations

The present study has several strengths including a state-of-the-art RNA-Seq approach and a common trauma in all participants. Nonetheless, our findings must be considered in the context of several limitations. First, gene expression analysis was performed in RNA-Seq on whole blood consisting of a mixture of different cell types. We adjusted for cell-type differences statistically; however, future work should examine the association in isolated cell types. Second, gene expression analysis was conducted using a cross-sectional design; thus, we cannot determine whether the observed associations with PCL are a consequence of the disorder or a part of its etiology. Third, participants were male responders to the WTC disaster, and while this helps to improve the biological heterogeneity of analyses, it is unknown how our results generalize to other traumatized samples or females. A longitudinal design across both genders is needed to determine the gender effect and direction of the association of gene expression with PCL. Fourth, we filtered out ~64% of the transcripts due to low counts in isoform-level analysis. The filtered transcripts may either not be expressed in our samples or not have been detected due to insufficient sequencing depth. Thus, a future direction for this research includes deeper sequencing to ascertain whether these filtered transcripts are truly not expressed and to replicate the results of the present study.

## Conclusions

The present study identified both shared and specific differential expression patterns at the gene and AS levels associated with total PTSD and its dimensions. This is the first study to characterize the landscape and relevance of AS in PTSD, and the results show that it is a promising direction for future studies. Inflammatory and metabolic pathways emerged as hypothesized, which may have implications in the treatment of PTSD. DE analysis associated with each dimension offers complementary findings, emphasizing the importance of studying the homogeneous components of PTSD.

## Supplementary information

Supplementary Materials

Supplementary Figure 1

Supplementary Figure 2

Supplementary Figure 3

Supplementary Figure 4

Supplementary Figure 5

Supplementary Figure 6

Supplementary Table 1

Supplementary Table 2

Supplementary Table 3

Supplementary Table 4

Supplementary Table 5

## Data Availability

The RNA-Seq data will be available at the Gene Expression Omnibus (accession number GSE164877) upon publication.

## References

[1] Kessler RC (2005). Lifetime prevalence and age-of-onset distributions of DSM-IV disorders in the National Comorbidity Survey Replication. Arch. Gen. Psychiatry.

[2] American Psychiatric Association. *Diagnostic and statistical manual of mental disorders: DSM-5*. 5th edn. (American Psychiatric Publishing:Washington, D.C, 2013).

[3] Steenkamp MM, Litz BT, Hoge CW, Marmar CR (2015). Psychotherapy for military-related PTSD: a review of randomized clinical trials. JAMA.

[4] Sareen J (2014). Posttraumatic stress disorder in adults: impact, comorbidity, risk factors, and treatment. Can. J. Psychiatry.

[5] Shalev A, Liberzon I, Marmar C (2017). Post-traumatic stress disorder. N. Engl. J. Med..

[6] Tylee DS (2015). Blood-based gene-expression biomarkers of post-traumatic stress disorder among deployed marines: a pilot study. Psychoneuroendocrinology.

[7] Koenen KC, Nugent NR, Amstadter AB (2008). Gene-environment interaction in posttraumatic stress disorder: review, strategy and new directions for future research. Eur. Arch. Psychiatry Clin. Neurosci..

[8] Afifi TO, Asmundson GJ, Taylor S, Jang KL (2010). The role of genes and environment on trauma exposure and posttraumatic stress disorder symptoms: a review of twin studies. Clin. Psychol. Rev..

[9] Nievergelt CM (2019). International meta-analysis of PTSD genome-wide association studies identifies sex- and ancestry-specific genetic risk loci. Nat. Commun..

[10] Atkinson, B. *Changes in eukaryotic gene expression in response to environmental stress*. (Elsevier, 2012).

[11] Glatt SJ (2013). Blood-based gene-expression predictors of PTSD risk and resilience among deployed marines: a pilot study. Am. J. Med. Genet. B Neuropsychiatr. Genet..

[12] Kuan PF (2017). Gene expression associated with PTSD in World Trade Center responders: an RNA sequencing study. Transl. Psychiatry.

[13] Breen MS (2015). Gene networks specific for innate immunity define post-traumatic stress disorder. Mol. Psychiatry.

[14] Breen MS (2018). PTSD blood transcriptome mega-analysis: shared inflammatory pathways across biological sex and modes of trauma. Neuropsychopharmacology.

[15] Dell’Osso L (2009). Brain-derived neurotrophic factor plasma levels in patients suffering from post-traumatic stress disorder. Prog. Neuro-Psychopharmacol. Biol. Psychiatry.

[16] Matsuoka Y, Nishi D, Noguchi H, Kim Y, Hashimoto K (2013). Longitudinal changes in serum brain-derived neurotrophic factor in accident survivors with posttraumatic stress disorder. Neuropsychobiology.

[17] Lambert WM (2013). Brain-derived neurotrophic factor signaling rewrites the glucocorticoid transcriptome via glucocorticoid receptor phosphorylation. Mol. Cell. Biol..

[18] Logue MW (2015). An analysis of gene expression in PTSD implicates genes involved in the glucocorticoid receptor pathway and neural responses to stress. Psychoneuroendocrinology.

[19] Kukurba KR, Montgomery SB (2015). RNA sequencing and analysis. Cold Spring Harb. Protoc..

[20] Alamancos GP, Pages A, Trincado JL, Bellora N, Eyras E (2015). Leveraging transcript quantification for fast computation of alternative splicing profiles. RNA.

[21] Grigoryev YA (2009). Genome-wide analysis of immune activation in human T and B cells reveals distinct classes of alternatively spliced genes. PLoS ONE.

[22] Ip JY (2007). Global analysis of alternative splicing during T-cell activation. RNA.

[23] Martinez NM (2012). Alternative splicing networks regulated by signaling in human T cells. RNA.

[24] King DW, Leskin GA, King LA, Weathers FW (1998). Confirmatory factor analysis of the clinician-administered PTSD Scale: evidence for the dimensionality of posttraumatic stress disorder. Psychological. Assess..

[25] Friedman MJ, Resick PA, Bryant RA, Brewin CR (2011). Considering PTSD for DSM-5. Depress. Anxiety.

[26] Stein, M. B. et al. Genomic characterization of posttraumatic stress disorder in a large US Military Veteran Sample. *bioRxiv* (2019) 10.1101/764001.

[27] Waszczuk, M. A. et al. Polygenic prediction of PTSD trajectories in 9/11 responders. *Psychological Med.* (2020) (in the press). 10.1017/S0033291720003839 PMC8186149 33092657

[28] von Kanel R (2007). Evidence for low-grade systemic proinflammatory activity in patients with posttraumatic stress disorder. J. Psychiatr. Res..

[29] O’Donovan A (2017). Current posttraumatic stress disorder and exaggerated threat sensitivity associated with elevated inflammation in the Mind Your Heart Study. Brain Behav. Immun..

[30] Michopoulos V (2015). Association of CRP genetic variation and CRP level with elevated PTSD symptoms and physiological responses in a civilian population with high levels of trauma. Am. J. Psychiatry.

[31] Watkins LE (2016). FKBP5 polymorphisms, childhood abuse, and PTSD symptoms: results from the National Health and Resilience in Veterans Study. Psychoneuroendocrinology.

[32] Mehta D (2018). Transcriptome analysis reveals novel genes and immune networks dysregulated in veterans with PTSD. Brain Behav. Immun..

[33] Dasaro, C. R. et al. Cohort profile: World Trade Center Health Program General Responder Cohort. *Int. J. Epidemiol.* (2015) 10.1093/ije/dyv099. 10.1093/ije/dyv099 PMC6074831 26094072

[34] Jansen R (2014). Sex differences in the human peripheral blood transcriptome. BMC Genomics.

[35] The PTSD Checklist (PCL): Reliability, validity, and diagnostic utility. *Proceedings of the annual convention of the International Society for traumatic stress studies* 1993. (International Society for Traumatic Stress Studies: San Antonio).

[36] Clouston SA (2016). Cognitive impairment among World Trade Center responders: long-term implications of re-experiencing the 9/11 terrorist attacks. Alzheimers Dement (Amst.).

[37] Ruggero CJ (2013). PTSD symptom dimensions and their relationship to functioning in World Trade Center responders. Psychiatry Res..

[38] Andrews, S. FastQC: a quality control tool for high throughput sequence data. Available online at: https://www.bioinformatics.babraham.ac.uk/projects/fastqc (2010).

[39] Martin M (2011). Cutadapt removes adapter sequences from high-throughput sequencing reads. EMBnet J..

[40] Langmead B, Salzberg SL (2012). Fast gapped-read alignment with Bowtie 2. Nat. Methods.

[41] Kim D (2013). TopHat2: accurate alignment of transcriptomes in the presence of insertions, deletions and gene fusions. Genome Biol..

[42] Pruitt KD, Tatusova T, Maglott DR (2007). NCBI reference sequences (RefSeq): a curated non-redundant sequence database of genomes, transcripts and proteins. Nucleic acids Res..

[43] Kent WJ (2002). The human genome browser at UCSC. Genome Res..

[44] Rosenbloom KR (2015). The UCSC genome browser database: 2015 update. Nucleic acids Res..

[45] Anders S, Pyl PT, Huber W (2015). HTSeq-a Python framework to work with high-throughput sequencing data. Bioinformatics.

[46] Patro, R., Duggal, G. & Kingsford, C. Salmon: accurate, versatile and ultrafast quantification from rna-seq data using lightweight-alignment. *bioRxiv* (2015) 10.1101/021592.

[47] Leek JT (2014). svaseq: removing batch effects and other unwanted noise from sequencing data. Nucleic Acids Res..

[48] Newman AM (2015). Robust enumeration of cell subsets from tissue expression profiles. Nat. Methods.

[49] Ren X, Kuan P-F (2020). Negative binomial additive model for RNA-Seq data analysis. BMC Bioinforma..

[50] Hastie, T. J. & Tibshirani, R. J. *Generalized additive models*, Vol. 43 (CRC press, 1990).

[51] Benjamini Y, Hochberg Y (1995). Controlling the false discovery rate: a practical and powerful approach to multiple testing. J. R. Stat. Soc. B.

[52] Faster k-medoids clustering: improving the PAM, CLARA, and CLARANS algorithms. *Proceedings of the International Conference on Similarity Search and Applications* (Springer, 2019).

[53] Tibshirani R, Walther G, Hastie T (2001). Estimating the number of clusters in a data set via the gap statistic. J. R. Stat. Soc.: Ser. B (Stat. Methodol.).

[54] Subramanian A (2005). Gene set enrichment analysis: a knowledge-based approach for interpreting genome-wide expression profiles. Proc. Natl Acad. Sci. U.S.A..

[55] Gene Ontology C. The Gene Ontology (GO) project in 2006. (2006). Nucleic. Acids Res..

[56] Ogata H (1999). KEGG: Kyoto encyclopedia of genes and genomes. Nucleic. Acids Res..

[57] Storey JD (2003). The positive false discovery rate: a Bayesian interpretation and the q-value. Ann. Stat..

[58] Langfelder P, Horvath S (2008). WGCNA: an R package for weighted correlation network analysis. BMC Bioinforma..

[59] Laaser I, Theis FJ, de Angelis MH, Kolb H-J, Adamski J (2011). Huge splicing frequency in human Y chromosomal UTY gene. Omics: a J. Integr. Biol..

[60] Powell ND (2013). Social stress up-regulates inflammatory gene expression in the leukocyte transcriptome via beta-adrenergic induction of myelopoiesis. Proc. Natl Acad. Sci. U.S.A..

[61] Luzzatto L, Notaro R (2002). Haemoglobin’s chaperone. Nature.

[62] Lever-van MilligenBA, Vogelzangs N, Smit JH, Penninx BW (2014). Hemoglobin levels in persons with depressive and/or anxiety disorders. J. Psychosom. Res..

[63] Vulser H (2016). Association between depression and anemia in otherwise healthy adults. Acta Psychiatr. Scandinavica.

[64] Shaw RJ (2009). LKB1 and AMP‐activated protein kinase control of mTOR signalling and growth. Acta physiologica..

[65] Rosenbaum S (2015). The prevalence and risk of metabolic syndrome and its components among people with posttraumatic stress disorder: a systematic review and meta-analysis. Metabolism.

[66] Wolf EJ (2016). Longitudinal associations between posttraumatic stress disorder and metabolic syndrome severity. Psychological Med..

[67] Rotival M, Quach H, Quintana-Murci L (2019). Defining the genetic and evolutionary architecture of alternative splicing in response to infection. Nat. Commun..

[68] Ergun A (2013). Differential splicing across immune system lineages. Proc. Natl Acad. Sci. U.S.A..

[69] Chapoval AI (2013). BTNL8, a butyrophilin-like molecule that costimulates the primary immune response. Mol. Immunol..

[70] Will CL, Lührmann R (2011). Spliceosome structure and function. Cold Spring Harb. Perspect. Biol..

