# Supplementary material for: Mapping the transcriptomics landscape of post-traumatic stress disorder symptom dimensions in World Trade Center responders
Source: Transl Psychiatry. 2021 May 24;11:310. doi: 10.1038/s41398-021-01431-6 (PMC8144574; doi:10.1038/s41398-021-01431-6)
Supplement: Supplementary file 1 — Supplementary Materials [file 41398_2021_1431_MOESM1_ESM.docx]

**Supplementary Methods**

*Weighted gene co-expression network analysis*

The weighted gene co-expression network analysis (WGCNA) ^1^ was performed to identify modules of correlated genes, isoforms and AS events on log transformed FPKM (gene), TPM (isoform) and PSI (AS), respectively. For each of gene, isoform and AS event WGCNA analysis, the Pearson correlation matrix was raised to the lowest power for which the scale-free topology fit index reached 0.8. The minimum module size was set as 30, and the cut-offs for splitting and merging modules were 2 and 0.1, respectively. A total of 20, 13 and 12 modules were identified from the gene, isoform and AS event WGCNA analysis, respectively. The expression profile for each module was represented by the eigen-gene, eigen-isoform and eigen-AS, respectively. The associations between module eigen-gene, eigen-isoform and eigen-AS with total PCL and each dimension were assessed using spline regressions ^2^, following adjustment for age, race, cell proportions, and potential surrogate variables. Significant modules were identified using p < 0.05.

At eigen-gene level, one module (salmon4) was associated with total PCL, re-experiencing, numbing and hyperarousal, whereas 4 modules were associated with avoidance. All but one showed nonlinear associations (Supplementary Table 4). At eigen-isoform level, 2, 2, 1, 3, and 2 modules were associated with total PCL, re-experiencing, avoidance, numbing and hyperarousal, respectively (Supplementary Table 4). On the other hand, at eigen-AS level, 3, 1, 0, 6 and 2 modules were associated with total PCL, re-experiencing, avoidance, numbing and hyperarousal, respectively (Supplementary Table 4). Supplementary Figure 5 compared the proportions of AS events among the significant module eigen-AS associated with total PCL and each dimension, respectively. Most of these modules showed nonlinear associations, consistent with the DE analysis result at genes, isoforms, and AS level. Spearman correlation coefficients computed for the estimated negative log p-values from spline regressions to summarize the global strength of module eigen-gene, eigen-isoform and eigen-AS associations among re-experiencing, avoidance, numbing, and hyperarousal were given in Supplementary Figure 6A, 6C and 6E, respectively, which indicated that analysis of avoidance had the lowest correlation as compared with that of the other dimensions. At different p-value thresholds, avoidance had the largest number of significant module eigen-gene (Supplementary Figure 6B), whereas analysis with module eigen-isoform were comparable across the different dimensions (Supplementary Figure 6D). On the other hand, hyperarousal and numbing had the largest number of significant module eigen-AS (Supplementary Figure 6F).

Bioconductor package clusterProfiler ^3^ was used to identify enriched KEGG pathways and gene ontologies among the genes in the four unique modules associated with total PCL or the dimensions from the eigen-gene analysis. The minimum and maximum gene set size were set as 15 and 500, respectively. The top 10 gene sets within each ontology for each module were reported in Supplementary Table 5. The skyblue2 module identified several immune related ontologies associated with neutrophil activation, whereas the lightpink4 module identified ontologies associated with interferon signaling pathway and viral response.

**Supplementary Table Legends**

**Supplementary Table 1.** List of DE genes at FDR < 0.05 for total PCL, re-experiencing, avoidance, numbing, and hyperarousal.

**Supplementary Table 2.** List of DE AS at FDR < 0.05 for total PCL, re-experiencing, avoidance, numbing, and hyperarousal.

**Supplementary Table 3.** List of GO and canonical pathway gene sets from GSEA at FDR < 0.1 or p-value < 0.001 for total PCL, re-experiencing, avoidance, numbing, and hyperarousal.

**Supplementary Table 4.** List of module eigen-gene, eigen-isoform and eigen-AS spline regression analysis for total PCL, re-experiencing, avoidance, numbing, and hyperarousal.

**Supplementary Table 5.** Top 10 KEGG pathways and gene ontologies (biological process (BP), cellular component (CC) and molecular function (MF)) for each of the four module associated with total PCL or the dimensions from the eigen-gene analysis.

**Supplementary Figure Legends**

**Supplementary Figure 1. A.** Number of clusters versus gap statistics. The optimal number of clusters was determined as 5. **B–F.** The 5 identified clusters. Within each cluster, the gray lines correspond to the estimated smooth functions of individual genes, whereas the red line corresponds to the mean estimated smooth function of the genes within the cluster.

**Supplementary Figure 2. A.** Pearson correlation coefficients among re-experiencing, avoidance, numbing and hyperarousal. **B.** Pearson correlation coefficients between PCL and estimated cell proportions.

**Supplementary Figure 3. A.** Pearson correlation coefficients comparing the negative log p-values among re-experiencing, avoidance, numbing, and hyperarousal from isoform-level analysis. **B.** Number of significant isoforms at different p-value thresholds from isoform-level analysis. **C.** Bar graph comparing the proportions of significant AS events associated with each dimension to the transcriptome-wide proportions of AS events detected by SUPPA. No AS events were detected for avoidance. **D.** Pearson correlation coefficients comparing the negative log p-values among re-experiencing, avoidance, numbing, and hyperarousal from AS-level analysis. **E.** Number of significant isoforms at different p-value thresholds from AS-level analysis.

**Supplementary Figure 4. A.** Pearson correlation coefficients comparing the negative log p-values among re-experiencing, avoidance, numbing, and hyperarousal from GSEA of GO gene sets. **B.** Pearson correlation coefficients comparing the negative log p-values among re-experiencing, avoidance, numbing, and hyperarousal from GSEA of canonical pathway gene sets.

**Supplementary Figure 5.** Bar graph comparing the proportions of AS events among the significant module eigen-AS associated with total PCL, re-experiencing, numbing and hyperarousal, respectively to the transcriptome-wide proportions of AS events detected by SUPPA. No eigen-AS was significantly associated with avoidance. A3: Alternative 3’ splice sites; A5: Alternative 5’ splice sites; AF: Alternative first exons; AL: Alternative last exons; MX: Mutually exclusive exons; RI: Retained introns; SE: Skipping exons.

**Supplementary Figure 6. A.** Pearson correlation coefficients comparing the negative log p-values among re-experiencing, avoidance, numbing, and hyperarousal from WGCNA module eigen-gene spline regression analysis. **B.** Number of significant modules at different p-value thresholds from WGCNA module eigen-gene spline regression analysis. **C.** Pearson correlation coefficients comparing the negative log p-values among re-experiencing, avoidance, numbing, and hyperarousal from WGCNA module eigen-isoform spline regression analysis. **D.** Number of significant modules at different p-value thresholds from WGCNA module eigen-isoform spline regression analysis. **E.** Pearson correlation coefficients comparing the negative log p-values among re-experiencing, avoidance, numbing, and hyperarousal from WGCNA module eigen-AS spline regression analysis. **F.** Number of significant modules at different p-value thresholds from WGCNA module eigen-AS spline regression analysis.

**References**

1. Langfelder P, Horvath S. WGCNA: an R package for weighted correlation network analysis. *BMC Bioinformatics* 2008; **9:** 559.

2. Hastie TJ, Tibshirani RJ. *Generalized additive models*, vol. 43. CRC press1990.

3. Yu G, Wang L-G, Han Y, He Q-Y. clusterProfiler: an R package for comparing biological themes among gene clusters. *Omics: a journal of integrative biology* 2012; **16**(5)**:** 284-287.
