# Supplementary figures and images for: Mapping the transcriptomics landscape of post-traumatic stress disorder symptom dimensions in World Trade Center responders
Source: Transl Psychiatry. 2021 May 24;11:310. doi: 10.1038/s41398-021-01431-6 (PMC8144574; doi:10.1038/s41398-021-01431-6)

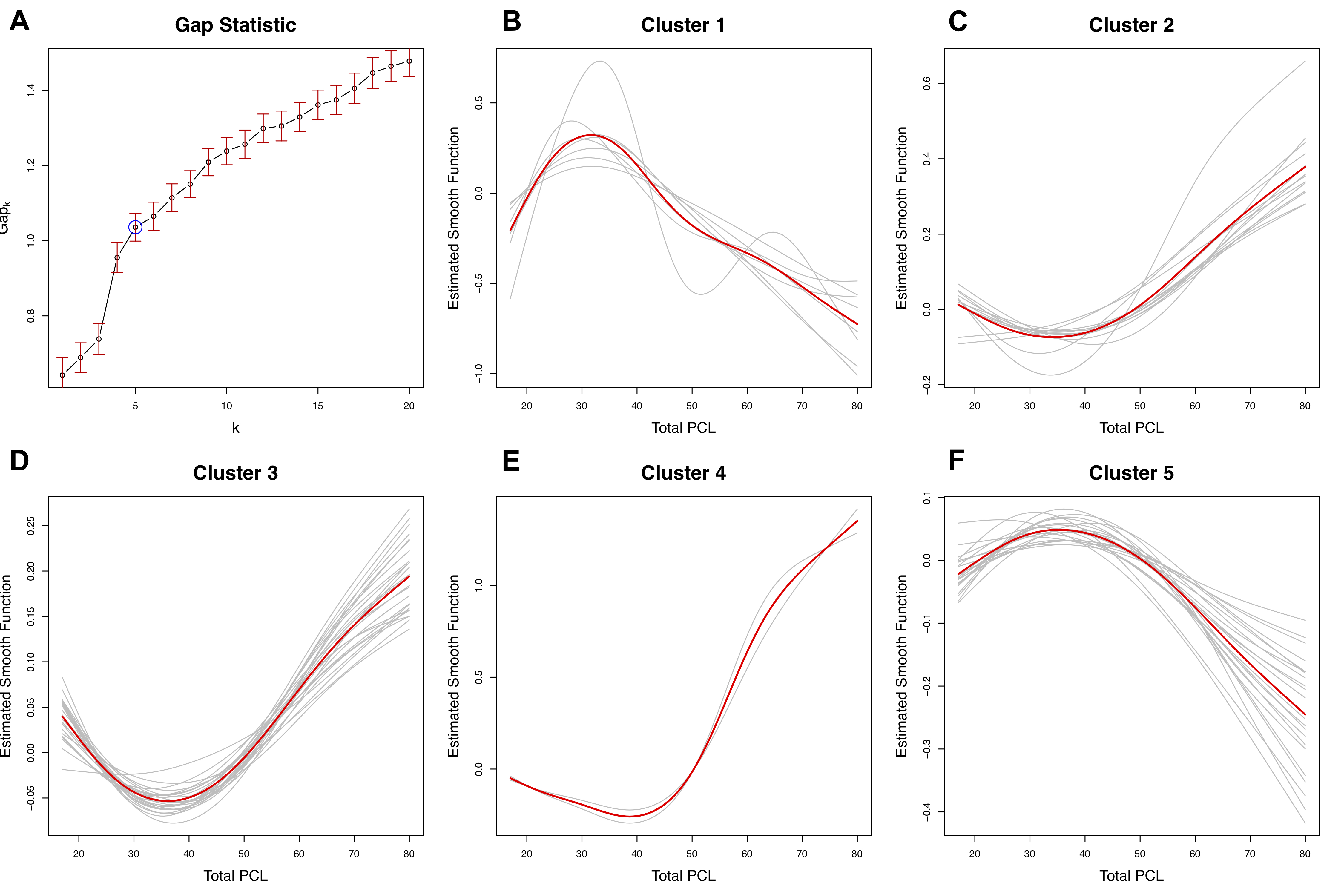

Supplement: Supplementary file 2 — Supplementary Figure 1 [file 41398_2021_1431_MOESM2_ESM.jpg]

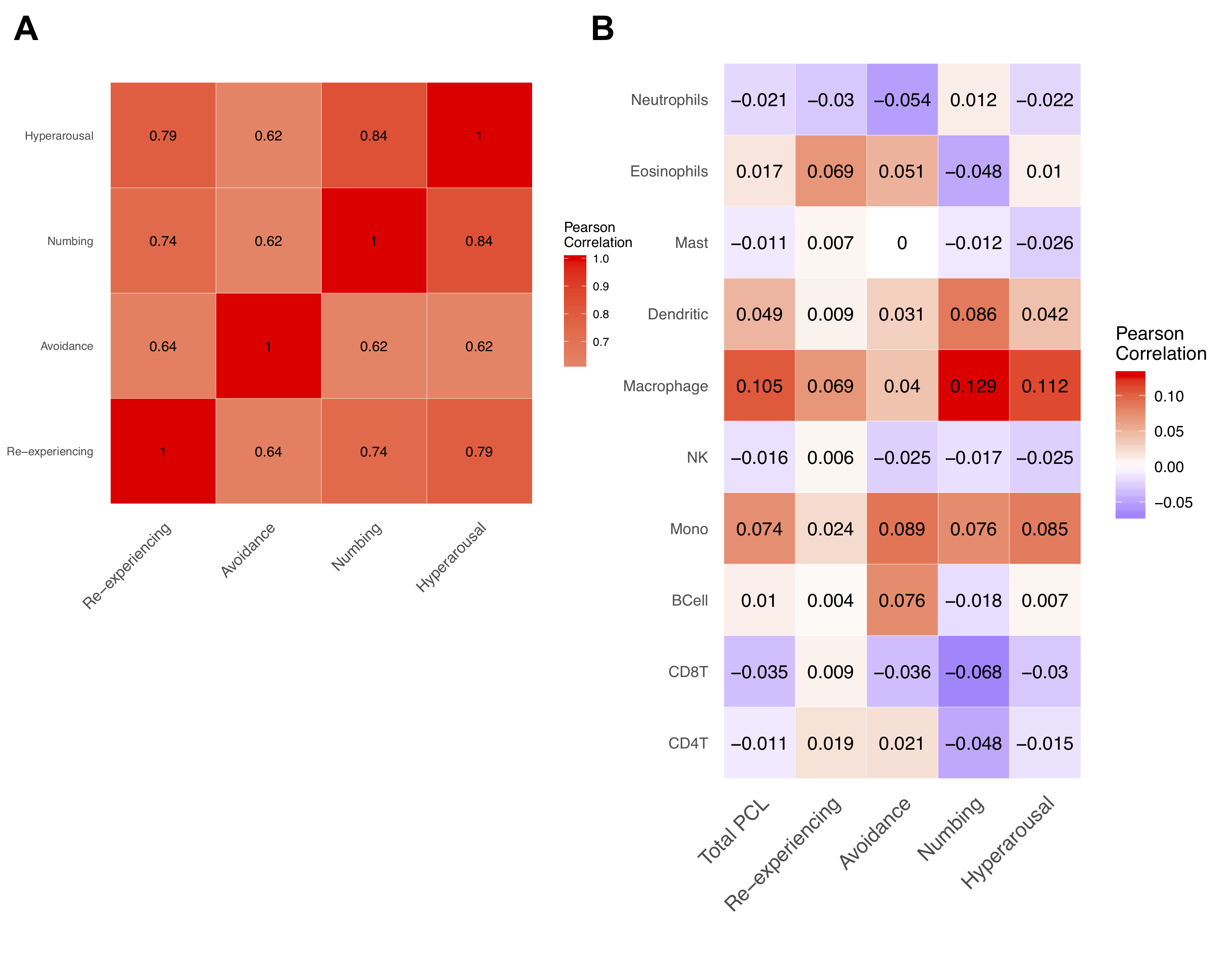

Supplement: Supplementary file 3 — Supplementary Figure 2 [file 41398_2021_1431_MOESM3_ESM.jpg]

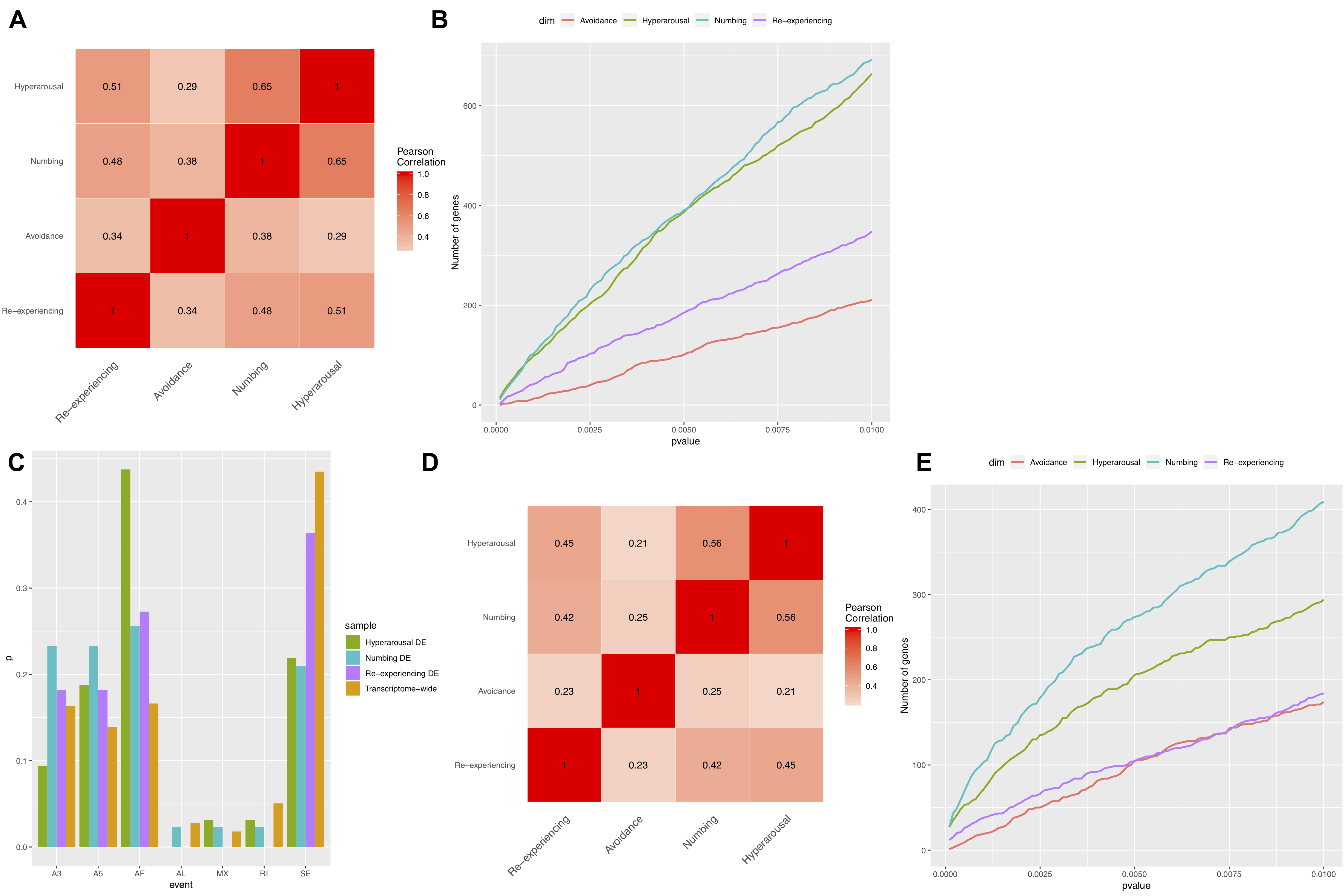

Supplement: Supplementary file 4 — Supplementary Figure 3 [file 41398_2021_1431_MOESM4_ESM.jpg]

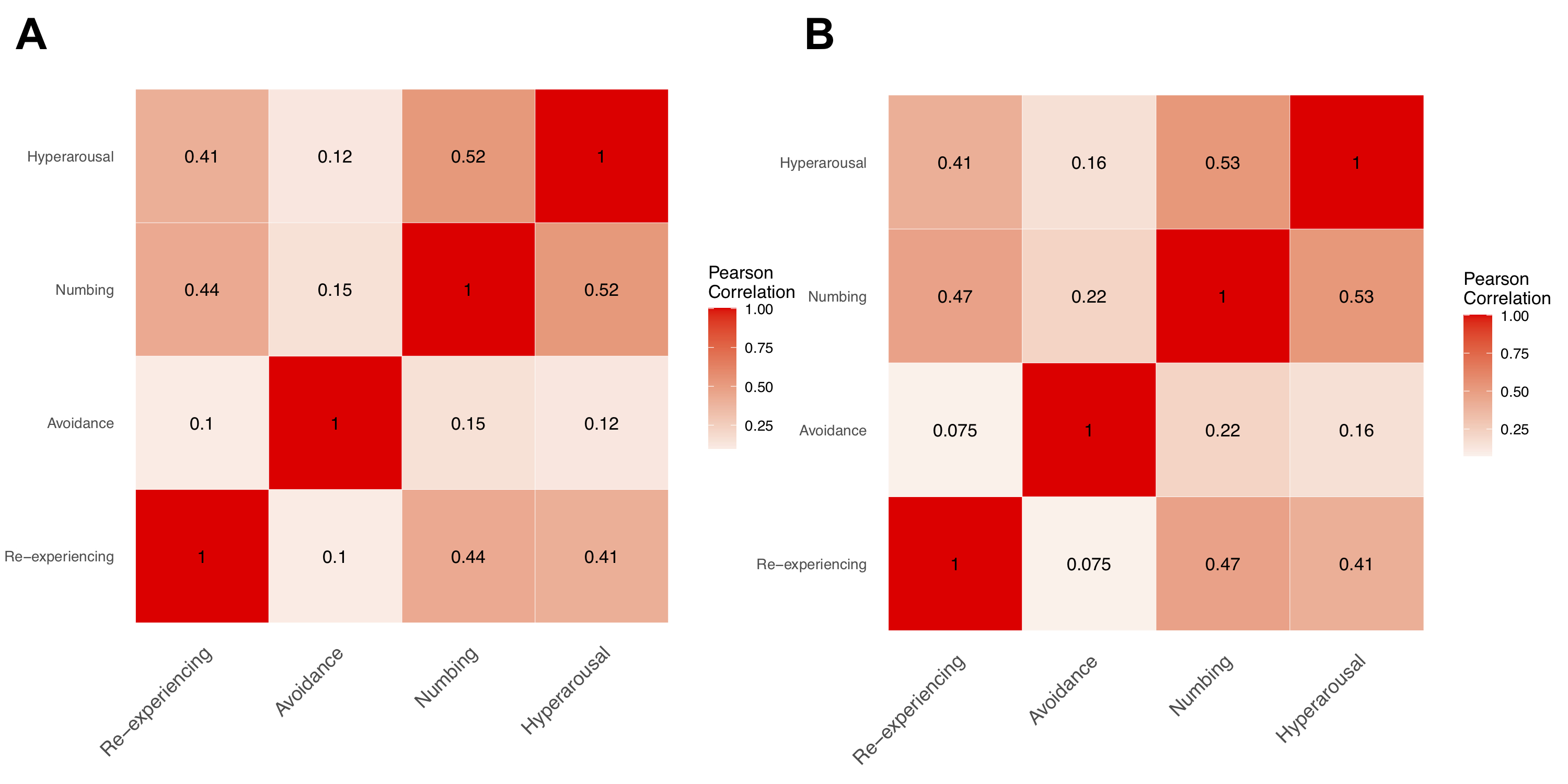

Supplement: Supplementary file 5 — Supplementary Figure 4 [file 41398_2021_1431_MOESM5_ESM.jpg]

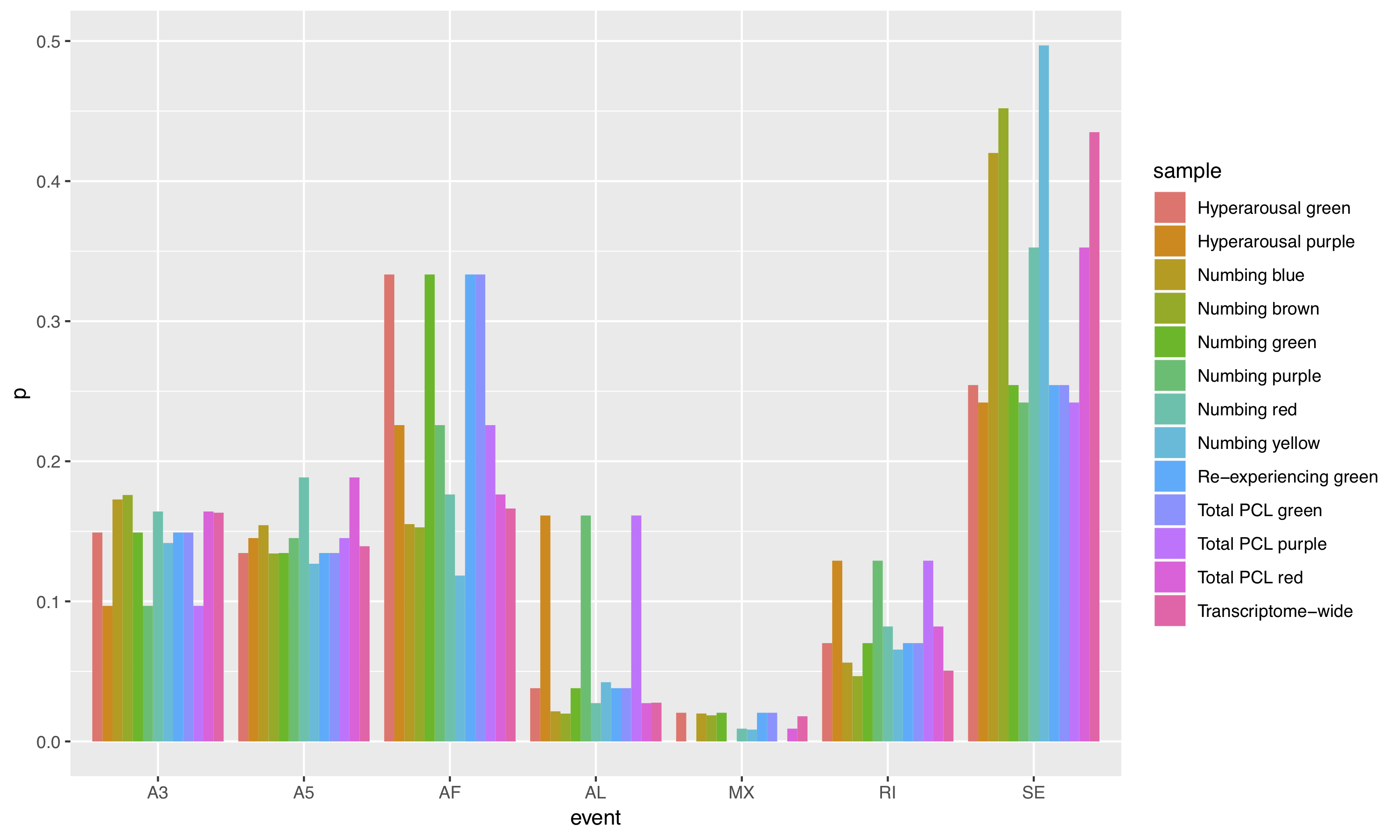

Supplement: Supplementary file 6 — Supplementary Figure 5 [file 41398_2021_1431_MOESM6_ESM.jpg]

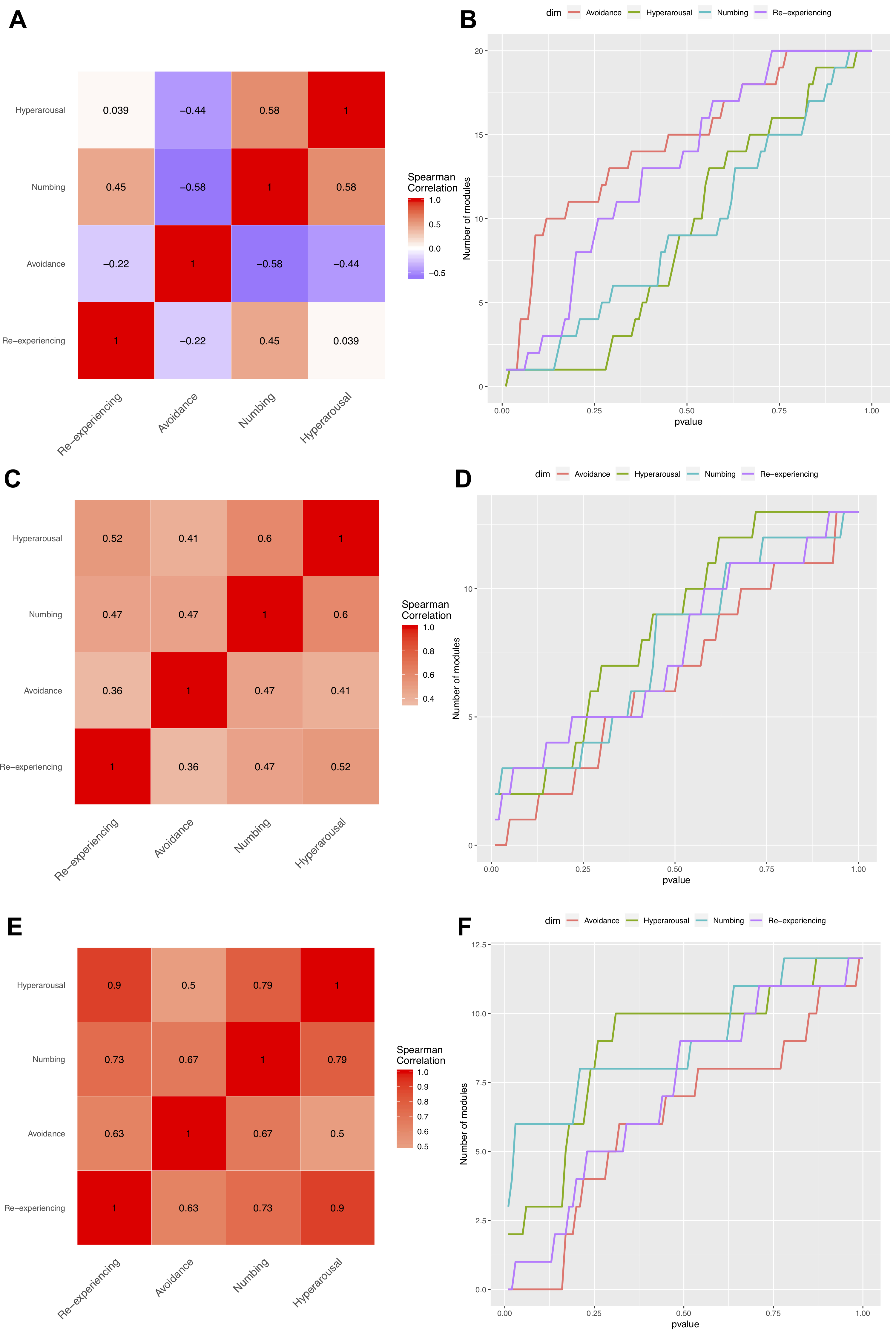

Supplement: Supplementary file 7 — Supplementary Figure 6 [file 41398_2021_1431_MOESM7_ESM.jpg]
